# Understanding physical activity participation in spinal cord injured populations: Three narrative types for consideration

**DOI:** 10.3402/qhw.v10.27295

**Published:** 2015-08-14

**Authors:** Anthony Papathomas, Toni L. Williams, Brett Smith

**Affiliations:** School of Sport, Exercise and Health Sciences, Loughborough University, Loughborough, UK

**Keywords:** Spinal cord injury, physical activity, rehabilitation, narrative, exercise medicine

## Abstract

The aim of this study was to identity the types of physical activity narratives drawn upon by active spinal injured people. More than 50 h of semi-structured life-story interview data, collected as part of larger interdisciplinary program of disability lifestyle research, was analysed for 30 physically active male and female spinal cord injury (SCI) participants. A structural narrative analysis of data identified three narrative types which people with SCI draw on: (1) exercise is restitution, (2) exercise is medicine, and (3) exercise is progressive redemption. These insights contribute new knowledge by adding a unique narrative perspective to existing cognitive understanding of physical activity behaviour in the spinal cord injured population. The implications of this narrative typology for developing effective positive behavioural change interventions are critically discussed. It is concluded that the identified narratives types may be constitutive, as well as reflective, of physical activity experiences and therefore may be a useful tool on which to base physical activity promotion initiatives.

The overarching aim of this paper is to adopt a unique narrative approach to better understand physical activity participation in the spinal cord injured population. Spinal cord injury (SCI) is a serious neurological condition most commonly caused by a traumatic force that bruises, partially ruptures, or completely severs the spinal cord. Spinal injured individuals may experience loss of mobility; complete paralysis; and impaired bowel, bladder, and sexual function at or below the site of injury (Gensel, 2014). Co-morbid physical consequences can include chronic pain; pressure sores; and obesity and its associated illnesses. From a psychological perspective, SCI can lead to elevated levels of depression and anxiety (Craig, Tran, & Middleton, 2009), as well as decreased self-esteem and increased social isolation (Geyh et al., 2012). The malaise is such that those with SCI often report lower quality of life and decreased life satisfaction compared to people without a spinal injury (Post & van Leeuwen, 2012). Overall, the sudden and significant changes brought about by SCI present an individual with numerous challenges.

Leisure-time physical activity (LTPA), defined as physical activity an individual engages in during their free time such as wheeling in the park, playing sport, or exercising in a gym (Martin Ginis et al., 2010), has been identified as a means to alleviate or prevent many of the physical and psychosocial health-related complications associated with SCI. For example, LTPA in SCI populations has been shown to reduce levels of perceived musculoskeletal and neuropathic pain (Norrbrink, Lindberg, Wahman, & Bjerkefors, 2012), correlate with better distribution of body fat (D'Oliveira et al., 2014), and lead to greater functional capacity such as ease of transfer (Martin Ginis, Jörgensen, & Stapleton, 2012). Participation in LTPA also correlates with better health on a more general level, with spinal injured people active for 25 min a day or more shown to display fewer cardiovascular disease and type 2 diabetes risk factors than their inactive counterparts (Buchholz, Martin Ginis, Bray, et al., 2009). With regards to mental health, recent reviews have described LTPA as an important determinant of subjective and psychological well-being post-SCI (Williams, Papathomas, & Smith, 2014), as well as improving overall quality of life (Tomasone, Wesch, Martin Ginis, & Noreau, 2013). Specifically, active spinal injured individuals have perceived LTPA to positively impact their self-esteem, self-confidence, psychological growth, and sense of purpose (Williams et al., 2014).

Despite the array of health benefits to be gained from regular LTPA, most spinal injured people live insufficiently active lifestyles. An estimated 50% are completely sedentary (Martin Ginis et al., 2010). The human cost of physical inactivity in SCI is great, as individuals unnecessarily endure acute and chronic health problems preventable through exercise (Gorgey, 2014). There is also an economic cost, with SCI healthcare across the lifetime calculated between 2.1 and 5.4 million dollars per person depending on age at injury and severity of injury (Cao, Chen, & DeVivo, 2011). Getting more spinal injured people physically active, then, would not only help lessen the so-called economic burden of SCI but more importantly, lead to healthier personal lives. As such, understanding SCI individuals’ motivation for LTPA is an important research agenda.

Traditionally, scholars have approached the issue of physical activity in spinal injured populations through the identification of perceived barriers, benefits, and facilitators (see Williams et al., 2014). In these studies, spinal injured participants are asked, via questionnaire or interview, to describe the factors they consider important in encouraging or preventing their engagement in LTPA. For example, Stephens, Neil, and Smith (2012) categorized perceived barriers to sport into organizational, medical, emotional, and informational factors, as well as perceived social stigma. Benefits were grouped into the themes relating to socialization, self-worth, physical challenge, and emotional gains. In a further study, the most prominent barriers to physical activity shortly after spinal unit discharge were cited as emotional distress, problems with self-care, and mental health problems (Vissers et al., 2008), with the most prominent facilitator identified as support from family, friends, and people in society. The authors also addressed barriers and facilitators 9 months post discharge and found that accessibility issues, physical health, and mental health were the primary barriers, with social and physical activity preparation within in the rehabilitation centre considered key to facilitating an active lifestyle. Approaching the subject from an “exercisers” versus “non-exercisers” perspective, Kehn and Kroll (2009) found that the active half of their spinal injured sample were facilitated by personal motivation, independence, availability of accessible facilities and personal assistants, fear of health complications, and weight management. On the other hand, those identified as inactive perceived a low return on physical investment, lack of accessible facilities, unaffordable equipment, no personal assistance, and fear of injury.

Although studies that explore physical activity barriers, benefits, and facilitators provide an important insight into the various personal, social, and environmental factors that impact on LTPA participation, an understanding of the motivational processes that lead to an individual becoming active or otherwise are largely ignored. There is little insight into why some individuals seek to overcome a given barrier to exercise; whereas others are halted in their tracks. Addressing this shortfall of process knowledge, a number of studies have called upon theoretical models of behaviour to better understand psychological predictors of LTPA in the SCI population. For example, the theory of planned behaviour (TPB; Ajzen, 1985), which asserts that behaviour is a function of an individual's intention to carry out that behaviour and perceived behavioural control, has proved relatively fruitful in explaining LTPA-related behaviours in SCI populations (e.g., Jaarsma, Geertzen, de Jong, Dijkstra, & Dekker, 2013; Latimer & Martin Ginis, 2005; Latimer, Martin Ginis, & Arbour, 2006). Similarly, social cognitive theory (SCT), and in particular self-efficacy for exercise, has been used to predict LTPA when spinal injured (e.g., Martin Ginis et al., 2011). Furthermore, the health action process approach (HAPA) has added an additional layer of insight into effective LTPA promotional strategies within the SCI community (Arbour-Nicitopoulos, Martin Ginis, & Latimer, 2009).

The discussed cognitive approaches to behavioural change, however popular, are not immune to critique. As an example, the validity and utility of the frequently deployed TPB has been questioned in recent years. It is argued that on the rare occasions studies have adopted longitudinal or experimental research designs, rather than simple cross-sectional correlations, there has been little support for the TPB (Sniehotta, Presseau, & Araújo-Soares, 2014). There is also an argument that the TPB explains insufficient variability of behaviour, with a recent review of more than 200 papers suggesting the figure just approached 20%, leaving the majority of health behaviour unaccounted for (McEachan, Conner, Taylor, & Lawton, 2011). Emphasising this point, in a sample of participants with a physical disability, the TPB construct of “intentions” predicted just 16% of physical activity behaviour (Kosma, Ellis, Cardinal, Bauer, & McCubbin, 2007). Even the HAPA, an integrative model that attempts to assimilate the best features of more dominant theories, has yet to demonstrate predictive efficacy (Armitage & Conner, 2000). Finally, given SCT's self-efficacy construct is accounted for in both TPB and HAPA, it can hardly be seen to resolve the issue of insufficient explanation of behaviour.

A possible explanation for the identified limitations of cognitive theories is that despite intentions leading to only minimal changes in behaviour (Webb & Sheeran, 2006), the role of rational, conscious, decision-making is overemphasised. In contrast, there is a growing appreciation of the role of non-conscious, implicit processes in behaviour change (Sheeran, Gollwitzer, & Bargh, 2013). The essential premise is that we don't necessarily weigh up the pros and cons of an action prior to engaging in it but rather act on influences outside of our awareness. For example, higher levels of physical activity have been associated with positive implicit attitudes and an attentional bias towards exercise cues (Calitri, Lowe, Eves, & Bennett, 2009). Models such as the TPB, SCT, and HAPA do not properly account for these potential non-reasoned influences on action.

Although adding a welcome dose of sophistication to the understanding of what determines a given health behaviour, implicit-based theories still prioritize cognitivism, albeit in a subconscious sense, over social and cultural factors. That is to say that even with the notion of rational reasoning downplayed, the primary root of a decision is still thought to reside somewhere deep within the actor's mind; a product of well-formed mental associations and cognitive biases. The limitation with this approach is that, ultimately, the psychological process is traced to the individual mind and, in doing so, the storied nature of human conduct is ignored (Sarbin, 1986).

Since the narrative turn of the 1980s, a growing number of scholars have theorized that we are all storytelling beings and that we make sense of our lives by emploting events into coherent narrative structures (Bruner, 1986; Jarvinen, 2001; Polkinghorne, 1988; Ricoeur, 1984). This process is not one that is traced to the individual mind, in which the story can be found inside the person, nor is it theorized as emerging from cognitions. Rather, our stories that we tell are drawn from narratives that are “outside us”; stories that are passed down from the social and cultural worlds that we are born into and which provide us with the templates for making sense of our experiences (Brockmeier, 2012).

Recently, narrative researchers, including those within sport (e.g., Smith, 2013), have also argued that narratives *do* things; they perform (Frank, 2010). Narratives are not simple representations of thoughts, emotions, and behaviours but are constitutive of these very things. Ultimately, narratives can shape what we think, how we behave, and what we imagine as possible. The stories we tell can open possible worlds, be powerful motivators of change, and determine decisions that lie ahead (Andrews, 2014; Brockmeier, 2009). As such, narrative appears to be an important, yet vastly overlooked, factor in determining health behaviours such as LTPA. The types of physical activity stories people tell of themselves can influence LTPA engagement and maintenance. But what types of stories do physically active people with SCI draw on from their sociocultural landscape and use when it comes to physical activity? This paper responds to this under-researched question. The aim of this study was to identity the types of physical activity narratives drawn upon by active spinal injured people.

## Methodology

### Narrative inquiry

Narrative inquiry refers to a psychosocial approach that focuses on stories. Although there are contrasting perspectives on narrative within the human sciences, what binds many together is the belief that human beings are meaning makers who, in order to interpret, show, and direct life, configure and constitute their experience using narratives that their social and cultural world has passed down (Brockmeier, 2012). The narrative inquiry practiced within this study adopts a relativist ontology and a subjectivist epistemology. That is to say that reality is believed to be multiple, socially constructed, and mind-dependent and that our route to knowledge is similarly value-laden (Smith & Deemer, 2000).

### Participants

University ethical approval was granted prior to adopting a purposive sampling procedure characteristic of qualitative research. Thirty UK-based participants with SCI, drawn from a larger project investigating the psychosocial health of people with spinal injuries, contributed to an extensive corpus of interview data. Participants ranged from 18 to 65 years of age, were between 1 and 28 years post injury, and were recruited via SCI-focused magazines, online discussion boards, and charities. All participants identified themselves as regularly physically active, with the terms “exerciser” or “sporty” frequently used. In accordance with spinal injury prevalence rates, there was a sex imbalance towards male participants with approximately 70% of the sample men (men/women, 3.8/1; see Wyndaele & Wyndaele, 2006).

### Data collection

In accordance with guidance from the university ethics committee, participants completed an informed consent form after one of the research team had explained the principal aims of the research project and offered an opportunity to ask questions. Participants then engaged in an open-ended, semi-structured interview with the first or second author at a location convenient to them. Interview length ranged from 60 to 170 min and the overarching focus throughout this time was participants’ physical activity experiences. Specifically, drawing on a loose and flexible interview guide, participants were asked to reflect on the nature of their involvement in physical activity both pre- and post- injury. Probing questions addressed barriers and facilitators to an active lifestyle, as well as the perceived benefits associated with LTPA. This structure was not allowed to inhibit participants discussing issues they felt were important and digressions were encouraged. As argued by Riessman (2008), “although we have particular paths we want to follow related to the substantive and theoretical foci of our studies, narrative interviewing necessitates following participants down their trails” (p. 24). Furthermore, to promote narrative data and the telling of stories, participants were regularly invited to “tell me a story about …” or “describe a time when …” For example, if a participant briefly mentioned enjoying physical activity, a narrative response was elicited by asking “can you tell me a story about when you particularly enjoyed being active?” Further probing questions, such as “how did that make you feel?” and “can you tell me more about that?” were used to encourage rich descriptive detail. On completion, interviews were transcribed verbatim and saved on a password protected computer.

### Narrative analysis

A structural analysis of narrative was conducted on interview data. Initially, to become familiar with the data and to gain an insight into how participants’ stories were structured, interview transcripts were repeatedly read through. The transcripts were annotated with conceptual comments concerning general thematic content (story) and the presence of particular plots that sequentially connect life events (narrative). The aim here was to identify a clear and persuasive narrative line—that is one that has a coherent narrative structure, that was repeated in different forms throughout the participant's account, and that was related to the issue of interest (i.e., LTPA). This represents an exploration of the “whats” of the narrative—what is its content and structure? (Gubrium & Holstein, 2009) With the narrative identified, attentions turned to the narrative resources that may have facilitated its construction. This process entailed inspecting the narrative data for clues as to how it was shaped. For example, how did the participant come to tell this story? How was the narrative accomplished on a social interactional level, both within described relationships and within the interview setting? Is the narrative one that circulates in wider society and therefore one that has been seen before? This represents an analysis of the “hows” of the narrative—how did it come to be constructed? (Gubrium & Holstein, 2009). Finally, identified narratives were named to reflect their thematic content, as well as their relationship to the broad sociocultural auspices from which they were constructed.

## Results and discussion

We provide a joint results and discussion section whereby participant quotes (pseudonyms used) are integrated with analytical interpretations. This combined approach allowed us to immediately contextualize and theorize the data as it is presented. Specifically, connections and differentiations between what participants said and the existing empirical knowledge-base can be made in a more transparent manner. Physically active spinal injured individuals drew from three principal narrative types: (1) exercise is restitution, (2) exercise is medicine, and (3) exercise is progressive redemption. These narratives types provide new knowledge as to how active spinal injured people understand their physical activity experiences; we now define each of these narrative types in turn.

### Exercise is restitution

The restitution narrative is a dominant storyline that projects hope for recovery after illness or injury (Frank, 2013). In SCI this has been translated to “yesterday I was able-bodied, today I'm disabled, but tomorrow I'll be able-bodied again” (Smith & Sparkes, 2005, p. 1096). The *exercise is restitution* narrative provides a subtle modification to this narrative type by specifically prescribing exercise, rather than medical science, as the chief means by which a person can return to their former, able body. The new narrative therefore reads, “Yesterday I was able-bodied, today I'm disabled, but tomorrow, through exercise, I'll be able-bodied again.” The term exercise is used, rather than LTPA, as it is the term used by the participants whose stories informed the narrative. Participants whose stories hung on this narrative structure described a continued engagement in LTPA and were often motivated to seek out regular and focused exercise opportunities:

I definitely wanted the opportunity to do exercises which would test whether or not I had any movement below what I perceive to be my injury level and what that movement was. So, for example, I'd always been told that I'd got biceps and wrist action, but since having gone to activity-based rehabilitation I have now discovered that I've got some core muscles that I'm able to use … I am really amazed at how much, not only movement and core strength I've gained, but also sensory sensations where in my legs and in my lower back and sort of bum muscles I can feel what felt like early feelings I had in my core muscles when I was doing exercise. Like slight tremors and tension building. So yeah, it's actually very exciting for me. I'm just taking it easy, and being very open minded, but I am quite pleasantly surprised. (Sarah, 44)

Sarah references her attendance at “Active Rehabilitation,” one of a small number of private rehabilitation centres in the UK that offer customized, high-intensity exercise programs, with a view to maximising physiological and neurological capacity. She goes on to construct a story whereby functional and sensory improvement is *emplotted* as a direct consequence of this exercise regime. With the exercise is restitution narrative reinforced, Sarah commits to continued exercise engagement and the additional restoration it might bring. This finding echoes previous work which has identified spinal injured participants who align with restitution as a key motivator to engage in LTPA (Perrier, Smith, & Latimer-Cheung, 2013).

Critically, Perrier et al. (2013) warn that an overt focus on restoration, although highly motivating, may narrow opportunities towards the recovery-centric types of exercise characteristic of active-rehabilitation initiatives at the expense of alternative, less outcome-directed activities. As such, those that tell an exercise is restitution story may be precluding themselves from a variety of other LTPA opportunities, the diversity of which may be psychosocially fulfilling and supportive of exercise maintenance over a long period of time. The following words from Thomas, a 47-year-old tetraplegic, illustrate a further potential complication with telling restitution-based narrative:

I'm a firm believer in never give up; you never know what's around the corner. Obviously I know you know it would be a miracle to get up and walk one day, just to even move my arms, get my hands working or something would be great …. So if I can keep the training up, and keep my body used to doing certain things then I'll always believe that something might happen. But if I didn't have that belief, I don't think I would want to be here really that would be the end of it. So, I have to have that belief. (Thomas, 47)

Driven by a desire to move again and restore his body to its former, pre-injured state, Thomas is highly motivated to “keep the training up” and continue his exercise regime. If the motivation to exercise is predominately inspired by the goal of recovery, as opposed to a goal of enjoyment or social interaction, then what happens when recovery is not forthcoming? The answer, for Thomas, is clear; he would lose all motivation for exercise and also perhaps, as is intimated, for life. What Frank (2013) might call the “chaos narrative” beckons, whereby life is without meaning and seen as never getting better. Given the permanency of SCI paralysis, this represents a troubling predicament for Thomas and his existing motivation must be viewed as fragile. In the absence of the “miracle” he describes, how long can he continue to buy into the exercise is restitution narrative? The psychological disruption that occurs when one's personal experience is misaligned with one's personal story (McLeod, 1997) is unlikely to facilitate continued LTPA engagement. These insights advance the field by illustrating narrative influences on a spinal injured person's motivation to be active. Knowledge of this exercise is restitution narrative can help sensitise the listener to the fact even a robust commitment to exercise on the surface, can be a fragile one beneath.

### Exercise is medicine

Exercise as a form of medicine is an emerging concept of growing popularity within academic, medical, and policy circles. Regular exercise can alleviate, as well as prevent, a myriad of existing physical (Warburton, Nicol, & Bredin, 2006) and psychological (Penedo & Dahn, 2005) maladies. So robust is the evidence for the medicinal qualities of exercise, it has even been described as “the much needed vaccine to prevent chronic disease and premature death” (Sallis, 2009, p. 3). The *exercise is medicine* narrative type for people with SCI reads as “I experience an ailment, then I engage in exercise, then the ailment is eased or eradicated.” The “ailment” may be minor or severe, physical or mental, but it is separate from the primary injury to the spine. In essence, it is a story of improved health and well-being whilst living with SCI rather than a story of cure. Robert, a 61 year old who sustained his injury 5 years previously, provided an example of the exercise is medicine narrative:

I really find when I go to the gym in the morning and my arms are stiff, my muscles are stiff, it's hard work to move, they hurt breathing and I struggle to get out of the car. I get into the chair and go to the gym and I'm like “oh dear.” After 2 or 3 weights I'm starting to loosen up. When I'm finished, pleasantly tired, very loose, tired but I can nearly leap into the car. All of those feelings of stiffness, the hurt is gone. My spasms are just about non-existent for a little while. And when you're incomplete like me, this is a big, big, thing. An awful lot of medication to attempt to keep them under control. But through exercise, and I'm a strong believer in that, I don't have any really severe spasms. (Robert, 61)

Robert's story tells of a visit to the gym for a weight training session. In the beginning he is tired, stiff, and in pain; he exercises, then returns to his car supple and reinvigorated. Robert even draws a favourable comparison between his exercise routine and his traditionally prescribed medication—the former considered more effective than the latter in controlling painful muscle spasms. Stories like this reinforce the link between exercise and health and encourage continued LTPA. As a story of health improvement, rather than a story of cure, the dangers associated with an exercise is restitution narrative are banished. First, health improvement is personal, relative, and multifaceted; unlike “cure” which is very much singular and absolute. Indeed, health improvement may well be constructed as being “less unwell.” The perceived “return on physical investment” then, an important LTPA motivator for spinal injured people (Kehn & Kroll, 2009), is much more likely to be considered satisfactory when expectations for exercise are not limited to outright recovery. Additionally, inherent within a goal of ongoing ailment relief and long-term health, is that exercise should also be *ongoing* and *long-term*. With no fixed endpoint outside of the actor's control—such as walking again—the risk of frustration and despondency is nullified. Robert, like other SCI people subscribing to an exercise is medicine narrative, interprets LTPA as a means to manage his condition; not a means to recover from it.

As well as symptom alleviation and health improvement, an exercise is medicine narrative also speaks to stories of physical preservation and illness prevention. For example Jon, a 65-year-old tetraplegic, stated:

It's maintenance. I know I'm not going to regain any more function. I've got all the function I'm going to get now. The important thing is to maintain that. Make sure it doesn't get worse. And that's the challenge as I get older. Like everybody else everybody ages … I know one tetraplegic who must be 70 now and he doesn't do as much as he used to. He used to be able to walk with crutches—which I can't do. But he doesn't do that very often and when I say “why not?,” he says “oh it's just an effort. My legs can't do it.” I have no idea when I get to 70 what I'm going to feel like. What's important to me is I want to be as independent as I am now and that I need to keep working at it. I've got this sort of feeling that if I don't, then it's going to be worse … so that's the sort of underlying motivation to keep doing things. (Jon, 65)

Jon's exercise is medicine narrative differs subtly to Robert's story in that it primarily addresses maintaining existing health and guarding against future decline in a bid to “age successfully”—that is age in the absence of disease and functional deterioration (see Bülow & Söderqvist, 2014). Jon's narrative therefore, might also be termed *exercise is “preventative” medicine*. Although his existing health is good, he is driven to continue regularly exercising in order to maintain health and to remain independent as he gets older. Staying physically active to improve quality of life in older adulthood is a much espoused medical narrative for which the evidence is strong (see Ashe et al., 2009). Whether seeking health improvement like Robert or health maintenance like Jon, the medicinal benefits of LTPA diminish quickly if engagement does not persist and so continued participation ensues. Those who buy into, and live their lives by, the exercise is medicine narrative then, develop a powerful commitment to living an active lifestyle and duly reap the benefits in terms of current and long-term health and well-being. For the SCI population, who typically experience a range of co-morbid health complications and whose independence may already be compromized, it is a very appealing and potentially very useful narrative type.

The exercise is medicine narrative may also pertain to the psychological benefits of an active lifestyle. Lucy, a 45-year-old paraplegic, expressed her understanding of the impact of exercise on her feelings of depression:

Yeah but for me it was the endorphin thing for me. I knew it would make me feel better and I know however I'm feeling, whether I'm angry or sad, it's the active meditation when I'm on my bike because it calms me down and then it's the euphoria afterwards. Getting off the bike and thinking that was good. For me it was get off your bike and feel better. I was depressed when I came out (of the rehabilitation unit). I wouldn't have agreed with that at the time but looking back now I wasn't my normal bouncy self and I knew my way out of that was exercise. Coming back to those chemicals for me, I knew if I'm not exercising … this is just going to be a downward spiral. (Lucy, 45)

Lucy's narrative is in tune with the finding that perceived mental benefits are a major LTPA facilitator for spinal cord injured populations (Williams et al., 2014). The narrative also echoes the psychological benefits of exercise literature where evidence is strongest for its role in the alleviation of depressive mood states, both clinical and subclinical (Biddle & Mutrie, 2007; Wolff et al., 2011). So powerful is this medical narrative in culture, Lucy is able to cite the release of “endorphins” during exercise as the proposed physiological mechanism that leads to reduced depression. Although the endorphin hypothesis is still very much a hypothesis (Dinas, Koutedakis, & Flouris, 2011), within public consciousness it is very much “a fact” and therefore provides Lucy with a sufficiently acceptable explanation of consequence to reinforce the exercise is medicine narrative plot. This furthers knowledge as it illustrates how culturally dominant narratives have the capacity to shape physical activity beliefs and behaviour. In terms of Lucy's motivation to exercise, the legitimacy of the endorphin hypothesis is less relevant that her own perception of its legitimacy; “narrative truth” overrides “scientific truth” when it comes to our thoughts and actions. If endorphins lend credibility to the exercise is medicine plot, they inspire belief in it, which in turn, promotes exercise behaviours.

### Exercise is progressive redemption

A *progressive* life-narrative is considered to be a rudimentary plot structure characterized by an upward trajectory of overcoming challenges towards some positive outcome of self-improvement or personal betterment (Gergen & Gergen, 1997). Building on this idea, McAdams and colleagues have coined the term *redemption narrative* which tells of a “transformation from a bad, affectively negative life scene to a subsequent good, affectively positive life scene. The bad is redeemed, salvaged, mitigated, or made better in light of the ensuing good” (McAdams, Reynolds, Lewis, Patten, & Bowman, 2001, p. 474). The notion of progressively overcoming difficult scenarios to arrive at some better place further down the line typified the stories of many of the physically active spinal injured participants in this study. In a particularly detailed example, Robert constructed his exercise experiences as very much informed by a progressive redemption narrative:

But they (cardiac nurses) did push you … they were trying to convince you that despite a heart problem you could still be active. That might have been the ammunition I had later when I became paralysed. That's what's pushed me to where I am. The heart attack I think was such an experience, and paralysis was another life changing experience, which at the time, I got over one so I got over the other one. Something inside of me, consciously or unconsciously, enabled me to become stronger for it. It feels that way now, I can go on even if the machinery is letting you down (laughs). I can still do more. I know I can. I know I can still do more … I cannot do nothing, I cannot sit and be inactive. I'll wheel up and down if I have to. I'll wheel up and down the house. Anything. Must do it. If there was anything to tell anybody about being in a wheelchair it's you must do something. You must enable yourself, you must empower yourself, you have to do it … I know for a fact everybody goes through a bad place. I know they do but I guarantee it, you do come out the other side. If you have the spirit and help from people, you do come out, I promise you. I'm living proof. If you saw what I've been through you'd think “why are you still here?” If you saw the misery I went through “why are you still here?,” … because you have to try …. (Robert, 61)

Robert draws parallels between two distinct stories; becoming active after a heart attack and becoming active after paralysis. The link is that both life events are interpreted through a redemption narrative—the movement from bad (illness/injury) to good (return to physical activity). The statement “I got over one so I got over the other one” suggests that the former provides a narrative map for success in the latter. Specifically, the redemption story that is told initially served as a *guiding story to live by* when later faced with the challenge of a SCI. Living by a redemption story is therefore not only a means to interpret experience, but it can also shape future experience. It fuels a desire to try for progression and “become stronger.” This commitment to succeed in the face of adversity is a useful commodity when pursuing an active lifestyle with SCI. Subscribing to the notion of *exercise is progressive redemption* then, may facilitate resilience against the many barriers to LTPA that people with SCI face. It therefore represents a potentially useful narrative resource for the promotion of physical activity in the spinal cord injured population. Indeed, Robert is keen to espouse the possible blanket relevance of this redemption plot when he states “everybody goes through a bad place” but “you do come out the other side.” For him, a redemption story should be the grand narrative for all people with SCI. It is a valid suggestion when placed within the context of research that demonstrates enhanced psychosocial well-being in those interpreting their lives through a progressive redemption narrative (McAdams et al., 2001).

As a narrative of the self, a progressive redemption story impacts on the teller's identity. The power of narratives to shape identity has been well articulated (see Holstein & Gubrium, 2000; McAdams, 1993; Smith & Sparkes, 2009), and the process is illustrated in the following reflections from Jon:

If your spinal cord is severed that's it forever. It's the only part of your body that doesn't regenerate. People obviously react in different ways to that. Obviously a common reaction is anger and “why me?” and depression and not wanting really to come to terms with it or be able to come to terms with it. I don't think I was indifferent in a lot of respects other than “it's done.” There's nothing I can do from what happened, now I have to focus on what I can do. I think that is very much, you know, people are either a glass half full or a glass half empty. I've always been a glass half full person. I've always tried to look at the positive side. So that kept me in good stead. Some people are just very negative about it. In fact I've seen a lot of people drop out of the gym. I was going for 5 years and you get to know people on the machine next to you and you talked to them. They come for 3 or 4 weeks or they get a GP (physician) referral for 6 to 8 weeks, never see them again. I am annoyed at what happened to me at the gym (loss of accessible equipment), mostly because it was avoidable and they should have done something about it. But it's like being in the wheelchair; I just get on and do something else. Life's not fair, no it's not but I'm not a natural quitter. I mean there are times when I don't feel like doing something—but that's rare. (Jon, 65)

In this extract Jon performs a progressive redemptive narrative. He does not dwell on his SCI, or the setback that comes when the accessible equipment in his gym is removed. Life is not fair but he remains upbeat and he emphasises his ability to simply “get on and do something else.” Ricoeur (1992) has argued that the identity of the story constructs the identity of the teller and Jon duly projects a self that is resilient, positive, and capable of coping with whatever life throws at him; including regularly exercising. Jon is a man who continues to move forward in the face of obstacles (progressive) and who can turn bad situations into good ones (redemption). It is an identity that brings a sense of pride and Jon is quick to emphasise his “glass half full” persona and he even distinguishes himself from other “very negative” spinal injured acquaintances who have been quick to give up their exercise regimes. Inspiring a valued self-identity presents a further useful quality of an exercise in progressive redemption narrative—to bow in the face of barriers becomes a slight on self, it questions ones identity. Better to struggle through difficult exercise barriers and be active, than to succumb and lose one's sense of self. This study is the first to link the broad life-narrative of progressive redemption to physical activity adherence. We have advanced the field by suggesting how an individual narratively interprets setbacks in a general sense impacts on how exercise barriers and setbacks are interpreted. Specifically, when the interpretation occurs through a progressive redemption lens, regular LTPA may be more likely.

## Final reflections

In this article, we extend traditional work on physical activity and sport by moving beyond a focus on cognitive models of behaviour change towards a narrative perspective. Three narrative types that circulate in culture and which people with SCI draw on were identified. Each narrative is similar in that all operate to help motivate people to become active and sustain a physically active lifestyle. In narrative terms, then, the three narratives *do things* in that each guides exercise behaviours. There were however differences and attended dangers between each. The exercise is restitution narrative was different to the exercise is medicine and exercise is progressive redemption narrative as it emphasised exercise as means to a cure for SCI and the teller was reliant on medical discoveries to successfully live by it. The exercise is medicine differed from the exercise is restitution narrative and progressive redemption narrative in that it was primarily concerned not with a medical cure but with using physical activity as a means to improve and sustain psychological and physical quality of life. In contrast, the exercise is progressive redemption narrative emphasised the transformative qualities of successfully overcoming exercise barriers to better the self. In this regard, the exercise is progressive redemption narrative is principally characterized by positive identity change, rather than an emphasis on the promotion of psychological well-being, physical health, or a cure from SCI.

Although distinct, the identified narrative types need not be told exclusively by an individual. Frank (2013) argues that it is possible for different, even opposing, narratives to be told alternately and also to coexist. This is dependent on the degree of narrative stability associated with the story told; to what extend does the teller buy into the story? (Papathomas & Lavallee, 2012). If for example, a spinal injured person is convinced that exercise will one day lead to cure, the exercise is restitution narrative can be seen as robust and stable and therefore unlikely to be wavered from. However, it may be that the exercise is medicine narrative can coexist with the exercise is restitution narrative in those who hope for a cure but do not necessarily have total faith in it. In essence, a less stable exercise is restitution narrative. A statement along the lines of “exercise might help me recover but even if it doesn't at least I'll be fit” holds elements of both exercise as restitution and exercise as medicine. In a practical sense, drawing from two narratives, represents a psychologically healthier scenario and one that better supports continued physical activity. As Smith and Sparkes (2009) argue, the more narratives available to us, the greater chance of finding one that fits our experience and works for us. For the physically active spinal injured narrator, it avoids putting all their eggs in one basket and allows for different understandings of the physical activity experience. If a cure does not come, the narrative simply shifts towards exercise as medicine and physical activity engagement persists—for its general health benefits. It would not be unusual for individuals, particularly those in the early stages of SCI where the extent of disability can be unknown, to move between these two narrative lines. Indeed, exploring when these narratives are told in relation to the time of injury represents an important part of putting these narrative types to the test. Specifically, future research may address whether narratives differ a year post injury compared to 5 years post injury? Similarly, do the narratives told change according to age, sex, and type of SCI?

Continuing with the notion of interrelated narrative types, as a subsidiary to a broad life-narrative, exercise is progressive redemption may coexist with either of the first two narratives or equally it may stand independently. For example, a SCI person may seek cure as a form of redemption and as such subscribes to both the exercise is restitution and exercise is progressive redemption narratives. Similarly, some SCI individuals may conceive of redemption as getting on with a healthy and active life—therefore subscribing to the exercise is redemption and exercise is medicine narratives. Last, the exercise is progressive redemption narrative may work exclusively as exercise is interpreted as bringing redemption in and of itself irrespective of its impact on health or recovery. For example, a spinal injured person may become active to prove to themselves, and others, that life is not over. Every time a barrier is overcome to be active, redemption is achieved and identity is constructed—the narrative is fulfilled.

In identifying three narrative types, the paper offers a typology for understanding what motivates people with SCI to become, and stay, physically active. Frank (2010) argued that typologies present numerous benefits for those who perform a given narrative and for those who listen. Typologies allow the teller to critically reflect on the narrative that guides them and the influence it has on their broader lives. For example, the person with SCI who narrates an exercise is restitution story may be prompted to consider alternative motivations for exercise (e.g., enjoyment, social interaction, achievement) if they are made aware of the fragile inflexibility of their current story. From a listener perspective, Frank suggests a narrative typology allows the essence of experience to be quickly identified amidst the messy, complexity that characterizes each varied individual story. It is therefore a valuable tool for those implementing physical activity interventions, for example motivational interviewing or GP exercise prescriptions, as it becomes easier for the practitioner-listener to categorize, digest, and act appropriately upon, the story of a teller. So an awareness of the dangers associated with an exercise is restitution story will assist a practitioner in ensuring such dangers don't materialize.

Each of our identified narrative types holds specific theoretical implications for positive behavioural change interventions. The first, exercise is restitution, serves as a powerful motivator for exercise but it is vulnerable in the long-term once, as is invariably the case, functional restoration plateaus and it becomes apparent that activity will not lead to cure. As such, practitioners, such as sport and exercise psychologists, physiotherapists, rehabilitation workers, SCI support staff, and other relevant health professionals should be wary of those who tell stories informed by this plot line. This is not to say the exercise is restitution narrative should always be avoided. In the early stages of rehabilitation, when the full extent of damage to the spinal cord is unknown, a commitment to pushing physical boundaries may well be useful. Any functional restoration that is possible will be supported by a positive attitude during physical rehabilitation. Nevertheless, promoting the exercise is medicine narrative as a viable alternative, is most likely best introduced sooner rather than later in order to avoid rapid deterioration in exercise motivation should the recovery that is hoped for not materialize. Indeed, the exercise is medicine narrative presents with few negative consequences and serves as a useful life-narrative to guide LTPA participation over the life-course. Practitioners must devise ways to inspire buy-in to this narrative type and consider ways to promote it as the default means to interpreting exercise as a spinal injured person. If there were to be any critique it is managing expectations as to what is expected of exercise as a form of medicine. The traditional conception of medicine is prescribed medication which often, for minimal effort, provides a desired effect on a given symptom. Exercise *does* require effort and the effects—for example on mood or spasm/pain relief—can be more transient than might be hoped for. To address this, the more stable benefits, such as cardiovascular health, weight management, and increased independence will need to be emphasised. Finally, exercise is progressive redemption may be a useful narrative that SCI people subscribe to at the same time as an exercise is medicine narrative. A sense of identity that is based on overcoming barriers—in this case exercise barriers—is essential given the array of barriers spinal cord injured people face. It is particularly important given the number of environmental barriers that are difficult to change short-term (e.g., facilities, transport, inaccessible open spaces). The utopia of a totally accessible society remains someway off and so the role of overcoming a barrier to engage in LTPA will need to be fulfilled, to a greater or lesser degree, by all spinal injured people at some point; adopting an exercise as progressive redemption narrative will ensure this process is less of a frustrating one.

An issue with all narrative theoretical work concerns what form do implications take in practice; in what settings might these narrative insights be deployed to encourage other spinal injured people to become, or remain, physically active? One area might be the growing role of narrative in health communication. Scholars have found that personal stories and testimonies of a given positive health behaviour are more likely to be absorbed than traditional forms of information giving such as leaflets (see Kreuter et al., 2010). Taking such findings into account, the narrative types documented here may be used as the basis for a more engaging form of health communication. Structuring a short story around an exercise is medicine narrative might serve a dual function of absorbing the listener/reader into the information presented but also providing a narrative map, a story to hold on to, of what an active lifestyle looks like. Before this however, we encourage academics researching spinal injured populations to substantiate the three narrative types we have suggested in this paper. Does the narrative typology hold up across different populations? Are there modified versions of the three narratives presented? More expansively, are there other narratives circulating in culture and impacting on the LTPA experiences of spinal injured people? Our typology is not meant to be definitive but rather provisional and in need of consideration.

In conclusion, the narrative structures physically active spinal cord injured people draw on to make sense of their exercise experiences provide a unique means to interpreting how and why some people with SCI manage to live active lifestyles despite the many obstacles in their way. The typology of three narrative types provides a useful tool for understanding the ways a limited number of stories that circulate within a culture shape exercise experiences. The narrative types can help tellers reflect on the power of their own stories, as well as educate them towards the potentialities of other stories that are out there. For practitioners, knowledge of these stories can facilitate an understanding of motivational processes, as well as provide a starting point for behavioural change interventions. Future research should look to explore and critique the presence of this typology in other spinal injured populations and consider its utility as vehicle for exercise promotion.
